# Validation of 3D EM Reconstructions: The Phantom in the Noise

**DOI:** 10.3934/biophy.2015.1.21

**Published:** 2015-03-23

**Authors:** J Bernard Heymann

**Affiliations:** National Institute of Arthritis and Musculoskeletal and Skin Diseases, National Institutes of Health, 50 South Dr, Bethesda, MD 20892, USA

**Keywords:** electron microscopy, 3D image processing, single particle analysis, gaussian noise, signal-to-noise ratio, structural biology, resolution

## Abstract

Validation is a necessity to trust the structures solved by electron microscopy by single particle techniques. The impressive achievements in single particle reconstruction fuel its expansion beyond a small community of image processing experts. This poses the risk of inappropriate data processing with dubious results. Nowhere is it more clearly illustrated than in the recovery of a reference density map from pure noise aligned to that map—a phantom in the noise. Appropriate use of existing validating methods such as resolution-limited alignment and the processing of independent data sets (“gold standard”) avoid this pitfall. However, these methods can be undermined by biases introduced in various subtle ways. How can we test that a map is a coherent structure present in the images selected from the micrographs? In stead of viewing the phantom emerging from noise as a cautionary tale, it should be used as a defining baseline. Any map is always recoverable from noise images, provided a sufficient number of images are aligned and used in reconstruction. However, with smaller numbers of images, the expected coherence in the real particle images should yield better reconstructions than equivalent numbers of noise or background images, even without masking or imposing resolution limits as potential biases. The validation test proposed is therefore a simple alignment of a limited number of micrograph and noise images against the final reconstruction as reference, demonstrating that the micrograph images yield a better reconstruction. I examine synthetic cases to relate the resolution of a reconstruction to the alignment error as a function of the signal-to-noise ratio. I also administered the test to real cases of publicly available data. Adopting such a test can aid the microscopist in assessing the usefulness of the micrographs taken before committing to lengthy processing with questionable outcomes.

## 1. Introduction

The biomedical community benefits enormously from fundamental knowledge of the structures of biomolecules and associated mechanisms of function. The validity of these structures are therefore of vital importance. While X-ray crystallography and NMR produced numerous structures, there remain large classes of molecular complexes unsuited to these methods. In recent years cryo-electron microscopy (cryoEM) advanced to the point where 3D reconstructions can be interpreted at near-atomic resolution [1]. This enticement together with the ubiquity of good software for processing electron micrographs and doing single particle analysis (SPA) is driving its adoption in many laboratories. This is good progress, but with the problem that in the rush to determine structures, the importance of appropriate image processing practices may be diluted. It is recognized with increased urgency that adequate validation methods are needed to accompany the maps derived from electron microscopy [2].

Two broad approaches to validation of single particle reconstructions aim at establishing the (i) coherency of information within a data set, and (ii) consistency with external sources of information. The latter may include other structural techniques such as X-ray crystallography and NMR, or biochemical assays providing information about composition and stoichiometry [2]. Here we are concerned with the former, where the aim is to justify the 3D structures calculated from electron micrographs. The main tool used to assess the quality and detail of a reconstruction is Fourier shell correlation (FSC) [3]. How this tool is used is of crucial importance to validation.

In a typical set of particle images from micrographs, it is assumed that the signal is that portion of the data that is consistent over all particles (for the moment ignoring heterogeneity). In reference-based alignment of the images, the signal is implicitly defined as that part of the image that agrees with the reference, and this is reflected in the FSC curve. Given the freedom alignment offers, even noise images will provide a non-random result, leading to reproduction of the reference structure [4]—a phantom from the noise. Current approaches to distinguishing real particle images from noise images are based on some form of cross-validation. As with the R-free measure in X-ray crystallography [5], the concept is that data not used for alignment is compared with the reference as an assessment of the validity. The two common techniques are resolution limitation imposed on projection-matching [6], and processing different subsets independently (“gold standard”) [2,7]. A third approach is to take tilt pairs of micrographs, expecting consistent angles of orientation between pairs of images of the same particles [8].

Imposing resolution limits on particle alignment was meant to access structural elements with the most promising alignment information [6,9]. The low resolution limit excludes low frequencies with little orientation information, while the high resolution limit cut out the high, noisy frequencies [6,10]. The added value in this approach is that the information not used in the alignment but recovered in the reconstruction provides a measure of validation. Aligning pure noise images only reproduce those frequencies explicitly used in alignment. A variant of this idea is to exclude a band of frequencies in the middle region during alignment, using it for validation of the subsequent reconstruction [11,12]. A suggested elaboration is to replace the high frequencies beyond the high resolution limit with noise and recalculate the reconstructions and FSC curve [13]. In current practice, the choice of high resolution limit for the alignment of particle images is routinely based on a high value cutoff of the FSC curve (typically 0.7 or 0.8). When the resolution reported at the 0.5 or 0.3 cutoff is significantly beyond the limit used for alignment, it supports the validity of the reconstruction.

The use of independent image sets is simply a form of repetition commonly used in science. Obtaining essentially the same reconstructions from data sets processed independently is an excellent indication of the validity of those maps. The current practice (the “gold standard” [2,7]) is to separate a data set into two subsets (typically by assigning alternating images to the two subsets). Each subset is aligned separately, starting from a very low resolution reference map. At each iteration, the reconstructions from the subsets are compared by FSC to assess similarity. This approach has been taken further by building an atomic model in a map from half of the data, and validating it against a map derived from the other half of the data [14].

A third validation method increasingly being adopted is tilt pair analysis [8]. The level to which the expected correspondence between tilt pairs of particle images is achieved is an indication of the validity of the resultant reconstruction. Tilt pairs also solves the problem of handedness, because the 2D nature of electron micrographs means that all chiral information has been lost in projection [15].

These validation approaches rely on the persons and software to adhere to good practices, presenting the community with a finished product. Even with the best of intentions and apparently following all the rules, there is still room for error. A recent controversy highlighted the issues and disagreements regardless of the use of validation methods mentioned above [4,16–19]. One answer is to have the data analyzed by an independent party [20]. A positive development is the launch of a service to make electron micrographs available through the EMDB (the EMPIAR initiative, http://www.ebi.ac.uk/pdbe/emdb/empiar/) [21]. However, re-analysis is a time-consuming, laborious endeavor that cannot be pursued in every case. In addition, an independent party needs to be capable, unbiased and motivated to produce a fair result.

I want to propose a validation test that is relatively simple to perform. Given a map from a current project or from the EMDB and a small subset of images used for it, the images are aligned to the map without masking or resolution limits, and compared to a reconstruction from the equivalent number of noise images aligned to the same map. The expectation is that the reconstructions from micrograph images should give better resolutions than noise-derived reconstructions. I show that the resolution of a reconstruction is a good reflection of the errors in alignment as a function of the signal-to-noise ratio (SNR). The validation test is then applied to three real cases with publicly available data. The simplicity of the concept means that it can easily be implemented in current SPA workflows.

## 2. Materials and Method

All data processing was done in Bsoft [9].

### 2.1. Synthetic data sets

Two structures were selected, the first is the asymmetric proteinase K (PK) (http://www.rcsb.org, PDB-3DYB [22]), and the second is the icosahedral lumazine synthase (LS) (http://www.rcsb.org, PDB-1NQX [23]). Both were converted to 3D density maps using the program *bsf* [9] with a pixel size of 1 Å and sizes of 80^3^ and 200^3^, respectively. Random projections were generate using *bproject* [9] and gaussian (white) noise was added using *brandom* [9] to give an imposed SNR (SNR_imp_) defined as the ratio of projection variance to noise variance.

### 2.2. Public data sets

Micrographs (82) of the keyhole limpet hemocyanin (KLH) taken on a CCD camera were obtained from NRAMM, The Scripps Research Institute, San Diego (http://nramm.scripps.edu, KLH data set 1). The reference used for KLH is EMD-1569 (http://www.emdatabank.org [24]). Particles and background images were manually picked for the KLH images (size 240^2^ at 2.2 Å/pixel). The CTF was automatically determined for each micrograph and the particle and background images corrected by phase flipping and baseline adjustment using program *bctf* [9].

Micrographs (20) of the Prochlorococcus cyanophage P-SSP7 taken on film were obtained from the NCMI, Baylor College of Medicine, Houston (http://ncmi.bcm.edu). The reference used for P-SSP7 is EMD-1713 (http://www.emdatabank.org [25]). Particles and background images were automatically picked for the P-SSP7 images with manual cleanup (size 576^2^ at 1.17 Å/pixel). The CTF was automatically determined for each micrograph and the particle and background images corrected by phase flipping and baseline adjustment.

A set of particle images (124478) from HIV glycoprotein (HIVGP) micrographs were obtained from the EMDB (http://www.emdatabank.org, empiar10008, [17], size 128^2^ at 1.49 Å/pixel). The reference used for HIVGP is EMD-5447 (http://www.emdatabank.org [18]), binned to size 128^3^ and used with both positive and negative contrast. The defocus values provided with the particle images were used to correct the CTF by phase flipping and baseline adjustment.

### 2.3. Image alignment

The images were aligned against the reference maps using the program *borient* [9] with a 1° angular search within the asymmetric unit and using all information up to the Nyquist frequency except where indicated otherwise (in other words, no resolution-limited or gold standard approaches were followed). Note that the maps for KLH, P-SSP7 and HIVGP obtained from the EMDB were heavily masked. Reconstructions were done using *breconstruct* [9], which uses an algorithm that integrates oversampled images as central sections in Fourier space. Resolution was estimated by Fourier shell correlation [3] at a cutoff of 0.5 (FSC_0.5_) using *bresolve* [9].

## 3. Results

### 3.1. Alignment errors and their correspondence to resolution

The resolution of a reconstruction is a function of the number of images contributing to it and the SNR of those images. The SNR is the major determinant of the alignment of the images, with errors increasing as the SNR decreases. The proposed validation method presumes that the resolution of the reconstruction is a fair reflection of the errors in alignment. Obtaining the errors in real cases is far from trivial (see [26,27] for efforts to estimate or model errors). To examine the correspondence between alignment error and reconstruction resolutions, synthetic cases were analyzed.

**Figure 1:**
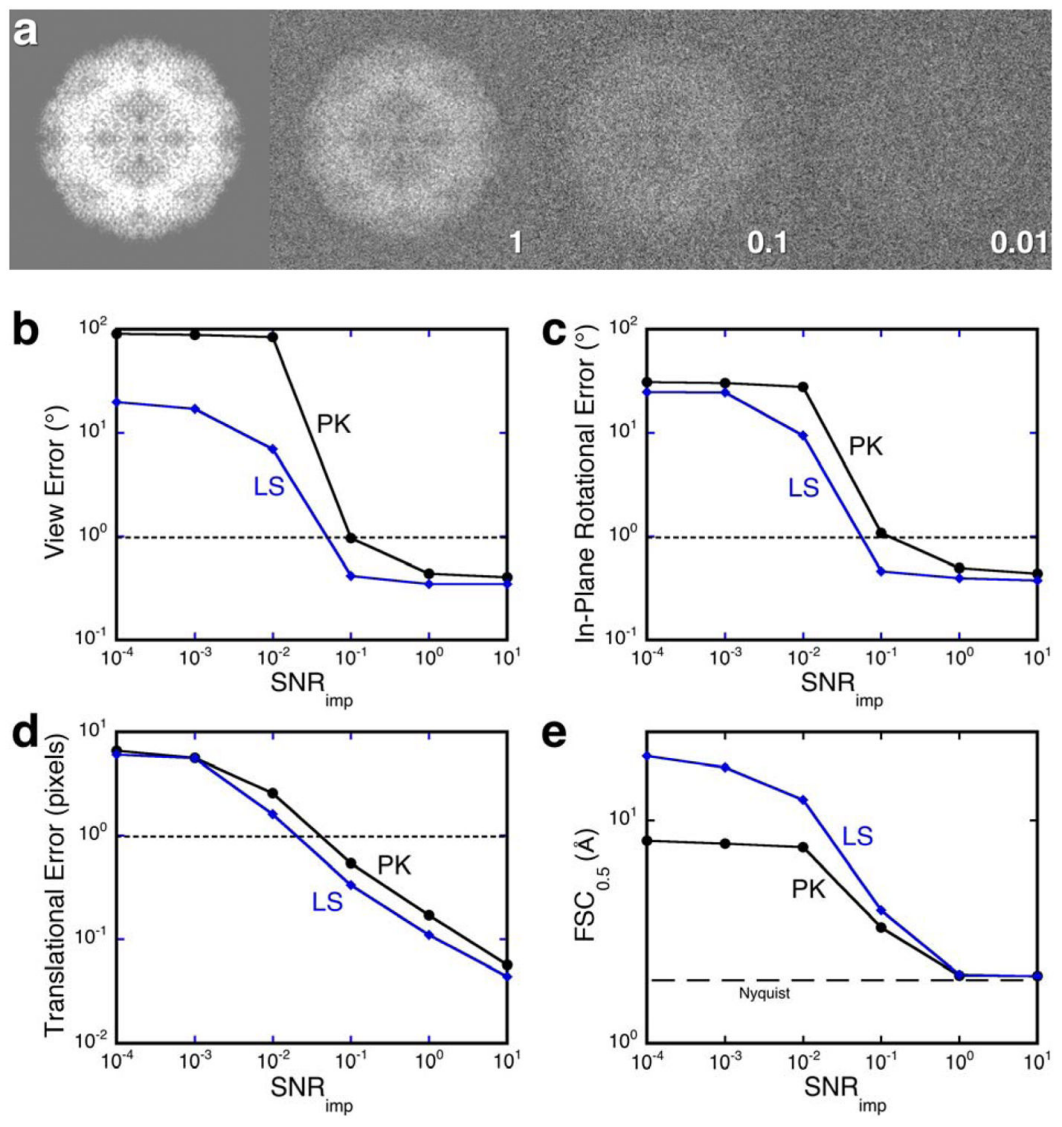
(a) A projection of lumazine synthase (left) corrupted with different levels of noise as indicated by the SNR_imp_ values (1–0.01). (b–d) The errors in alignment were determined for projections of synthetic maps of proteinase K (PK; black disks) and lumazine synthase (LS; blue diamonds) with different imposed SNR values. View (b) and in-plane (c) rotational errors show a rapid change between SNR values of 0.01 and 0.1. (d) Translational errors show a gradual change with the SNR. (e) Resolution estimates of reconstructions of PK from 5000 images, and LS from 100 images (6000 asymmetric units).

Two synthetic cases were chosen with different sizes and symmetries. Proteinase K (PK) is a 30 kDa monomer with no symmetry [22], while lumazine synthase (LS) is a 1.03 MDa 60-mer with icosahedral symmetry [23]. Density maps of these were calculated from the crystal structures, randomly projected, gaussian noise added to the projections (Figure 1a), and the resultant images aligned to the density maps using all information up to Nyquist frequency (2 Å in these cases). The measure of resolution chosen is the threshold of 0.5 in the FSC curve (FSC_0.5_), both as a conservative choice and because it is the most commonly reported in the EMDB (http://www.ebi.ac.uk/pdbe/emdb/).

The average errors in translation and orientation (view within the asymmetric unit and in-plane rotation) were calculated. Figure 1b–d shows the expected monotonic relationship with the SNR, but with distinct differences between translational and rotational errors. The translational errors (Figure 1d) increase steadily with a decrease in SNR. The plateau at very low SNR is due to an 8 pixel limit on the allowed extent of translation. In contrast, the rotational errors (Figure 1b, c) increase sharply with a decrease in SNR from 0.1 to 0.01. This indicates that rotational alignment is much more sensitive to noise than translational alignment. Similar trends have been shown by Joyeux and Penczek [28].

The point at which noise becomes intolerable depends on what is an acceptable error. For rotational errors, the threshold is chosen as 1° (dotted lines in Figure 1b, c), the angular step used in alignment. The corresponding SNR is ~0.10 for PK and ~0.09 for LS. An acceptable error for translation may be one pixel, indicated by a dotted line in Figure 1d. For PK the corresponding SNR is ~0.08 and for LS ~0.05. The differences between PK and LS could be due to size and symmetry, although other subtle factors including the structure factor distribution may also contribute.

Figure 1e shows the FSC_0.5_ estimations for reconstructions from a reasonable number of images in each case at different SNR’s. The resolution loosely follows the shape of the rotational error curves in Figure 1b, c, following a trend that is strongly influenced by the accuracy of rotational alignment. The resolution estimates are of course a function of the number of images, whereas the errors are not. The errors at an SNR of 0.1 are still acceptable, and an increase in the number images used in the reconstructions should improve the resolution. The exact SNR at which a particular set of images will transition from producing a valid reconstruction to one just reflecting the reference is likely case-specific. Nevertheless, the conclusion is that the particular SNR values that affect alignment translates well to the resolution estimates, and that the latter can be used as indirect assessment of how well the images can be aligned.

### 3.2. Reconstructions from limited numbers of images emphasize alignment differences

While the alignment errors in a synthetic case provides some idea of the influence of noise, examining the reconstructions resulting from the alignment is closer to the way SPA is practiced in real cases. The resolutions of the reconstructions are both dependent on the SNR of the images and their number. At very large numbers of images, the distinctions due to different SNR’s disappear as other limits are reached (such as microscope imaging envelopes and eventually Nyquist frequency). Such distinctions are more evident at smaller numbers of images and allow an estimation of the influence of alignment errors on reconstruction.

**Figure 2:**
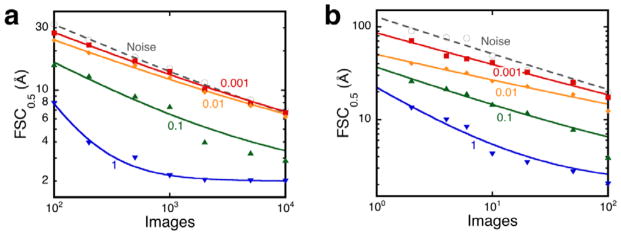
Resolution estimates (FSC_0.5_ values) for reconstructions from projections of synthetic maps with different imposed SNR values (numbers next to lines). (a) Proteinase K: size 80^2^ at 1 Å/pixel. (b) Lumazine synthase: size 200^2^ at 1 Å/pixel, icosahedral. Also shown are the resolution estimates of reconstructions from aligned pure noise images (open circles and dashed lines). Each point is the average of resolution estimates of 10 reconstructions.

To illustrate this, the projection alignment and reconstruction experiments were done as before on the two synthetic cases, PK and LS, varying the SNR as well as the number of images used in each reconstruction. A number of particles were selected randomly and two maps calculated from mutually exclusive halfsets, and the resolution estimated. Each estimate reported is the average from 10 random image selections. These maps were compared with reconstructions calculated from aligned noise images as control (Figure 2).

It is quite evident that as the SNR decreases, the resolution estimates exhibit an asymptotic approach to those for the pure noise images (Figure 2). There is a gradual transition from aligning to the object in the images to aligning noise. At some point the SNR is low enough that one cannot have confidence that the reconstruction is an accurate representation of the desired particle. The particular SNR at which the curves coincide with those of the noise images differ somewhat between the two cases. The main reason for this is that the SNR is not a well-defined parameter and is influenced by the size and structural information of the object in the images.

**Figure 3:**
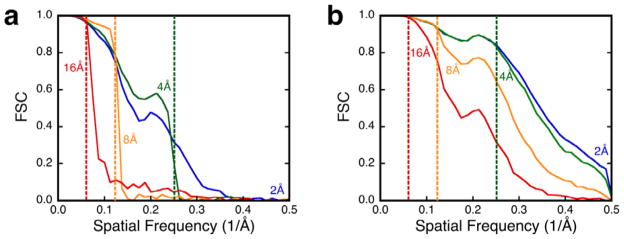
Resolution-limited alignment of proteinase K for SNR values of (a) 0.01 and (b) 0.1 show the difference between dominance of noise and alignable images. The dotted lines indicate the different resolution limits imposed: blue, 2 Å; green, 4 Å; orange, 8 Å; red, 16 Å. Images used: 10^4^.

The next question is: How close should the resolution estimates be to those of noise-derived reconstructions to be judged unacceptable? It is clear in the case of PK (Figure 2a) that the images with an SNR of 0.01 yield reconstructions very similar to those from noise. To shed more light on the matter, images of PK were aligned using different resolution limits and the reconstructions calculated. Figure 3a shows that an SNR of 0.01 precludes recovery of significant information beyond the alignment limit. In contrast, images with an SNR of 0.1 produce reconstructions with information extending towards the Nyquist frequency (Figure 3b).

It is quite clear that defining the SNR where the transition from aligning structure to aligning noise occurs is complicated and case-specific. The use of the synthetic cases illuminates some of the issues that need to be supported with real micrograph images.

### 3.3. Real cases with alignable images

In the synthetic cases, everything is known and set up in a well-defined manner. Nevertheless, the transition from aligning structure to aligning noise is ill-defined. A real case presents further complications: the exact orientations and origins of the particle images are unknown and errors cannot be determined accurately. Another problem is that the estimation of the SNR of the images is non-trivial and may not have the same interpretation as in synthetic cases. While the structural parts of the objects of interest are likely very well modeled, the nature of the imaging details and noise inherent in real images may not be readily reproduced. Therefore, in the two cases presented here, both particle and background noise images were extracted from the micrographs for analysis. The contrast transfer function (CTF) was corrected by phase-flipping.

The first case is a public data set of the keyhole limpet hemocyanin (KLH) collected on a CCD camera [29]. KLH is a cylindrical 7.8 MDa didecamer (20-mer) with D5 symmetry showing clearly distinguishable top and side views in the micrographs. The second case is the cyanophage P-SSP7, a T7-like podovirus of the cyanobacterium Prochlorococcus [25]. Only the reconstruction of the icosahedral capsid shell of 16.6 MDa is considered here.

**Figure 4:**
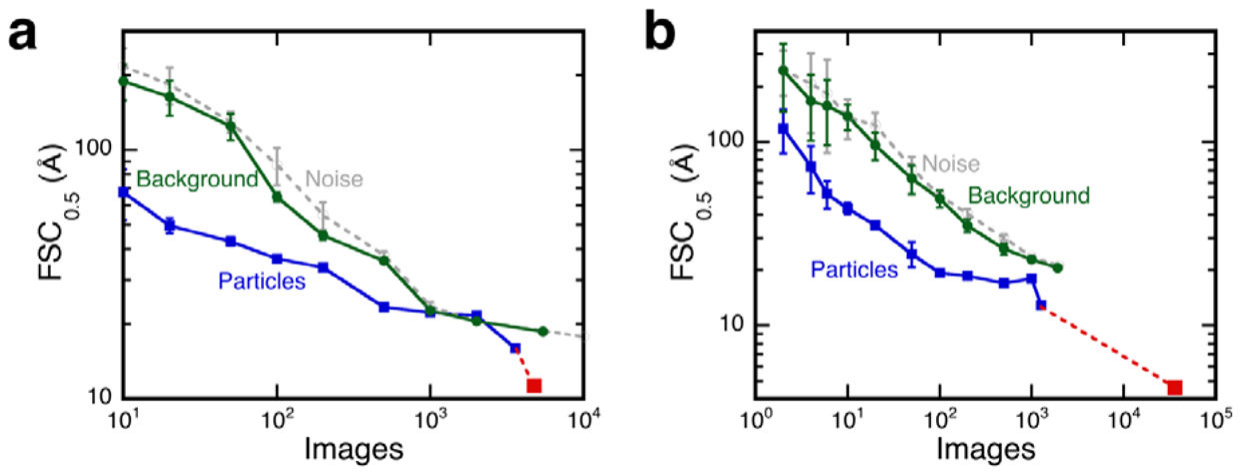
Resolution estimates (FSC_0.5_ values) for reconstructions from microscopic images (blue) for (a) KLH: size 240^2^ at 2.2 Å/pixel, symmetry D5, and (b) P-SSP7: size 576^2^ at 1.17 Å/pixel, icosahedral. The published resolutions are shown in red. Also shown are resolution estimates for aligned gaussian noise (gray) and background images (green). Each point is the average of resolution estimates of 10 reconstructions with standard deviations as indicated by the error bars.

The resolution estimates for reconstructions from particle images are much better than those for noise images from the same number of images (Figure 4). Of note, the behavior of micrograph background and gaussian noise images are very similar, suggesting that the noise images are reasonable substitutes for the background (maybe not surprising [30]). Towards larger numbers, the curves converge in both cases, and then diverge again. This is a reflection of the orientational information in the structure and is highly case-specific. The resolutions for the final KLH and P-SSP7 structures are significantly different from that of the corresponding noise-derived reconstructions. These are examples where the validity of the reconstructions is not in doubt.

### 3.4. Controversial case with questionable alignability

**Figure 5:**
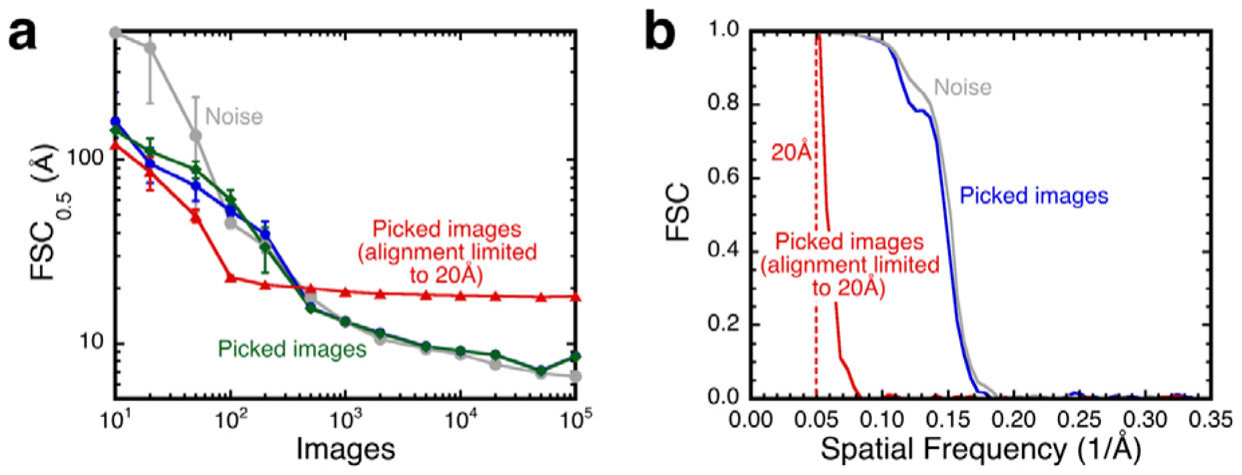
(a) Reconstructions using images from the HIVGP micrographs (blue disks and green diamonds) compared with those from gaussian noise images (gray disks). Both positive (blue disks) and negative (green diamonds) density references were tested because the contrast direction was not evident from the images. When the alignment of the images was limited to 20 Å, the resolution estimation did not improve beyond 18 Å (red triangles). Each point is the average of resolution estimates of 10 reconstructions with standard deviations as indicated by the error bars. The images were of size 128^2^ at 1.49 Å/pixel, symmetry C3. (b) FSC curves for the reconstructions from 10^5^ micrograph (blue) and noise (gray) images aligned to Nyquist, and micrograph images aligned to 20 Å (red).

A study reporting a structure for the HIV glycoprotein (HIVGP) generated a large amount of controversy [4,16–19]. The data for this study has now been released, and the boxed images were obtained from the EMDB. The first problem was that the images appear not to contain any recognizable structures (as noted in [19]), and the contrast direction in the images was unclear. The images were therefore aligned with both a positive and negative version of the final reconstruction [18]. A similar alignment of noise images produced reconstruction resolution estimates tracking those of the particle images aligned with both references, suggesting that they are indistinguishable from noise (Figure 5a). The only difference is at low numbers of images (< 100), where the noise-derived reconstructions show worse resolutions compared to the particle images. This may be due to the amplitude decrease with spatial frequency in the micrograph data, whereas the generated noise images have constant average amplitude over all frequencies. The FSC curves for reconstructions from both micrograph and noise images are indistinguishable but consistent with the claimed ~6 Å resolution (Figure 5b). The conclusion is that the images do not exhibit any alignable information as demonstrated in the other two cases.

To confirm this outcome, the micrograph images of the HIVGP were aligned using a resolution limit of 20 Å. If there is information beyond this limit and up to the 6 Å claimed [18], this should be clearly evident. The triangles in Figure 5a show that the maximum resolution obtained was 18 Å, even with a large number of images (the inverted reference map gave the same curve). The FSC curve (Figure 5b) also showed a sharp drop-off at this resolution, indicative of the alignment of noise. This reinforces the conclusion that these images are not distinguishable from noise.

## 4. Discussion

### 4.1. Validation by expecting a better reconstruction than from noise

In SPA, there are two questions validation needs to answer. Firstly, is the reconstruction an acceptable representation of the data? The traditional approach has been to compare projections from the reconstruction with raw particle images. Resolution-limited and gold standard approaches assess the coherence in the images in a more quantitative manner. Secondly, when a particular resolution is claimed for a reconstruction, is it justified? Pushing the processing to achieve higher resolutions may run the risk of increasingly incorporating noise, also known as “overfitting”. This becomes more problematic when near-atomic resolution is reported with the associated molecular modeling.

Here I propose a validation method that checks whether the achieved resolution is better than what can be expected from an equivalent number of noise images. The expected resolution of a reconstruction is embodied in the following well-accepted relationship between the spectral SNR (SSNR) of the reconstruction from N images, and the average SSNR of the images (adapted from [10,31,32]):

$$\mathrm{SSNR}(s,N)=\frac{N}{Cs}\mathrm{SSNR}(s)e^{-\frac{B}{2}s^{2}}$$

where *s* is the spatial frequency, C is a proportionality constant, and B relates to the alignment error. This requires three pieces of information: the number of images, the SSNR of the raw images, and the error in alignment. The alignment error is some (poorly defined) function of the SSNR of the images (as shown in Figure 1b–d). The resolution of reconstructions for synthetic cases tracks the alignment error (Figure 1e), suggesting that it can be used as an indirect way to reflect the SSNR of the images. The proposed validation test therefore plots the resolution against the number of images as a stand-in for the SSNR of the original images. When this plot is not significantly different from one generated form noise or background images, it is taken as indicating insufficient SSNR in the raw images to produce a valid reconstruction.

The analysis proposed is very simple and straightforward. The examples presented here are based on the algorithms employed in Bsoft [9] and may differ in detail when done in other packages. However, the basic algorithms are very well worked out standard approaches and should compare well in performance to other packages. The only inputs required are the final reconstruction and the images contributing to it. Masking was deliberately excluded, except for the already masked reference maps obtained from the EMDB. The orientation search is a grid-based polar Fourier method (modeled after Baker and Cheng [6]) with a standard correlation coefficient derived from a cross-correlation map. The polar resampling of the images for projection-matching has been shown to produce smaller errors than other methods [28]. No resolution-limits were used and no masking was done. No effort was made to obtain the best possible reconstructions, as that was not the goal. Nevertheless, the images aligned to the EMDB reference maps in both the KLH and P-SSP7 cases, produced better reconstructions than noise images (Figure 4). Indeed, the quality of the maps was consistent with the published maps, but just from fewer images. This demonstrates that the coherence in the images can be tested even in the presence of considerable high frequency noise.

At a typical SNR for good cryo-electron micrographs (~0.1 [33]), the particle images are alignable and valid reconstructions can be calculated. When the SNR decreases, as is expected for smaller particles and taking micrographs closer to focus, the validity of the reconstructions becomes more problematic. The case illustrated in Figure 5 is particularly disturbing, because the images do not contain readily recognizable densities that could be taken as representing the particles. One could argue that with better algorithms or more carefully selected parameters than were used here, a coherent structure could be observed in the images. However, the images and the reference map were those supplied by the authors using their processing pipeline [18]. The expectation is that at least there should be evidence of a correlation between the images and the map at low resolution. Figure 5 clearly shows that, when the alignment was resolution-limited (thus removing a large amount of high frequency noise), no significant information could be recovered beyond this limit. It is highly unlikely that the fundamentally sound algorithms used here would show no trace of a coherent structure that would be recoverable by another technique. The conclusion is that the SNR is so low that it cannot be distinguished from noise and no valid reconstruction can be generated (as noted by Henderson, 2013 [16]).

### 4.2. The number of images contributing to a reconstruction

It is common practice to collect large numbers of images and process them all at once. This runs the risk of aligning noisy images without considering an expected resolution that would be better than with noise images. A prudent way would be to collect a smaller number of images and make sure that valid reconstructions can be generated from them, even at low resolution.

Several studies examined the relationship between the number of particles and the reconstruction resolution [34–38]. Unfortunately, in none of these cases a comparison with the alignment of noise or background images was done. Stagg et al. presented an experiment where the introduction of errors decreased the calculated resolution [38]. However, completely randomizing alignment parameters should tend to the expected curve for uncorrelated noise. This is not the same as aligning to noise and is unlikely to distinguish coherent particles from noise. In another experiment, they refined particle images against correct and incorrect reference maps. While the final reconstructions were of the same resolution, at lower numbers of images the correct and incorrect alignments could be clearly distinguished. At least in this case there was an effort to target the specific coherence between the images.

Estimating the number of images to achieve a desired resolution is still an unsolved problem due to the uncertainties in SSNR and alignment error. Attempts to determine the SSNR are involved [33] and may not be practical for every case. A bigger uncertainty lies in the alignment error, where the combination of rotational and translational errors (Figure 1) are not easily modeled [27]. In general the plots between the numbers of images and the achieved resolution have been used to produce a global temperature factor combining the influence of SSNR decay and alignment errors [10]. In the current state of knowledge, each case needs to be explored on its own merits.

### 4.3. The practical value of limited-number reconstructions

Apart from its value in validation, an exercise such as that presented in Figure 2 is also an aid the image processor. First, the quality of micrographs can be judged fairly rapidly without the need to acquire large numbers. Because the SSNR is the key property determining the alignability, the microscopist can optimize data acquisition to provide the best interpretable images (see Cardone et al. [39] for a good application along these lines). Secondly, the image processor can judge whether steps such as particle picking and alignment produce acceptable results, adjusting parameters as needed. This may be faster than doing an exhaustive analysis (as done in Stagg et al. [37]). Thirdly, software developers can incorporate the trends in their programs to improve results. Fourthly, the final reconstruction can be judged as reasonable when limited-number reconstructions show better coherence than noise-derived reconstructions, and valid when it in addition complies with other criteria. Finally, it improves the understanding of the number of particle images required to achieve a desired resolution, and that an excessive number may increase the risk of questionable reconstructions.

### 4.4. Combining validation approaches

The validation proposed is not meant to be used in an iterative fashion as is typically done during SPA processing. Instead, it asks the question of whether a given reconstruction (e.g., from the EMDB) could have been derived from noise, given the number of images used. The proper use of resolution-limited (e.g., Figure 3) and gold standard approaches imposes stronger controls on the alignment and should in general yield valid maps. However, both these can be subverted by injudicious choices, such as using shaped masks, including high frequency noise in the resolution-limited approach, and violating the information independence between subsets in the gold standard approach (such as by pre-selecting subsets of data). It is therefore important to verify that the reported results conform to an expected outcome for the number of images used.

All three validation approaches can be combined in workflows. A data set of images can be divided into two or more subsets and each processed independently. Within each of these processing pipelines, resolution limits can be employed to limit alignment to only those spatial frequencies with strong and useful orientation information. Reconstructions can be generated from multiple selections from the images to produce plots of the resolution improvement with the number of images. At the same time, noise images can be aligned to the reference maps and reconstructions generated from different numbers of images. Comparison between the reconstructions from different subsets now offers a wealth of information about variability and validity.

## 5. Conclusion

The generation of a phantom reconstruction from aligned noise is here proposed as a baseline to assess the validity of a reconstruction from micrograph images of presumably identical particles. This is both a sanity check for the microscopist at an early stage in a project, as well as for reconstructions already deposited in the EMDB. The ease with which it can be incorporated into current workflows for processing electron micrographs should make it a valuable addition to the validation toolbox.

## Abbreviations

EM: electron microscopy
EMDB: electron microscopy databank
SPA: single particle analysis
FSC: Fourier shell correlation
SNR: signal-to-noise ratio
SSNR: spectral SNR
